# Accelerated Chemical Thermodynamics of Uranium Extraction from Seawater by Plant‐Mimetic Transpiration

**DOI:** 10.1002/advs.202102250

**Published:** 2021-10-28

**Authors:** Ning Wang, Xuemei Zhao, Jiawen Wang, Bingjie Yan, Shunxi Wen, Jiacheng Zhang, Ke Lin, Hui Wang, Tao Liu, Zhenzhong Liu, Chunxin Ma, Jianbao Li, Yihui Yuan

**Affiliations:** ^1^ State Key Laboratory of Marine Resource Utilization in South China Sea Hainan University Haikou 570228 P. R. China; ^2^ Research Institute of Zhejiang University‐Taizhou Taizhou 318000 P. R. China

**Keywords:** biomimetic, directional‐channel hydrogel, seawater, uranium extraction

## Abstract

The extraction of uranium from seawater, which is an abundant resource, has attracted considerable attention as a viable form of energy‐resource acquisition. The two critical factors for boosting the chemical thermodynamics of uranium extraction from seawater are the availability of sufficient amounts of uranyl ions for supply to adsorbents and increased interaction temperatures. However, current approaches only rely on the free diffusion of uranyl ions from seawater to the functional groups within adsorbents, which largely limits the uranium extraction capacity. Herein, inspired by the mechanism of plant transpiration, a plant‐mimetic directional‐channel poly(amidoxime) (DC‐PAO) hydrogel is designed to enhance the uranium extraction efficiency via the active pumping of uranyl ions into the adsorbent. Compared with the original PAO hydrogel without plant‐mimetic transpiration, the uranium extraction capacity of the DC‐PAO hydrogel increases by 79.33% in natural seawater and affords the fastest reported uranium extraction average rate of 0.917 mg g^−1^ d^−1^ among the most state‐of‐the‐art amidoxime group‐based adsorbents, along with a high adsorption capacity of 6.42 mg g^−1^ within 7 d. The results indicate that the proposed method can enhance the efficiency of solar‐transpiration‐based uranium extraction from seawater, particularly in terms of reducing costs and saving processing time.

## Graphical abstract

A plant‐mimetic directional‐channel hydrogel is designed for transpiration‐enhanced uranium extraction from seawater. Attributing to the transpiration boosted seawater transport and the raise of the temperature, the chemical thermodynamics for uranium adsorption is enhanced and the uranium extraction capacity is increased by 79.33%.

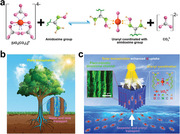

## 1 Introduction

The sustainable development of humanity is strongly dependent on sustainable access to resources, particularly energy and water.^[^ ^1^ ^]^ In this context, because of the rapid increase in the consumption of terrestrial resources, resource acquisition using non‐traditional approaches has become an urgent need.^[^ ^2^ ^]^ Meanwhile, it is known that seawater is a reservoir of diverse resources, especially in the form of large water reserves and abundant uranium deposits.^[^ ^3^ ^]^ Global seawater is estimated to contain 4.5 billion tons of uranium, and the reserve amount is 1000 times more than that in terrestrial uranium ores.^[^ ^1^, ^4^ ^]^ Thus, the sustainable extraction of uranium from seawater is considered a promising approach for the long‐term sustainable development of nuclear power. Many types of uranium adsorbents have been developed for the efficient recovery of uranium from seawater,^[^ ^5^ ^]^ including inorganic materials,^[^ ^6^ ^]^ synthetic organic molecules/polymers,^[^ ^7^ ^]^ natural or modified protein/biomass‐based macromolecules,^[^ ^8^ ^]^ and various types of nanostructured adsorbents such as grafted polymeric porous supports, metal–organic frameworks (MOFs),^[^ ^9^ ^]^ covalent–organic frameworks (COFs),^[^ ^10^ ^]^ porous carbons,^[^ ^11^ ^]^ porous aromatic frameworks (PAFs),^[^ ^12^ ^]^ and porous organic polymers (POPs).^[^ ^13^ ^]^ However, a large number of technical difficulties are associated with the processing of the massive quantities of seawater required for uranium extraction because of the extremely low uranium concentration (3.3 ppb) in seawater.^[^ ^5a^ ^]^

In the extraction of uranium from seawater, the functional groups present in the adsorbent and the temperature around the adsorbent are two critical factors that affect the uranium adsorption efficiency.^[^ ^14^ ^]^ According to the law of mass action (1) and the Arrheniusformula (2)

(1) $$v\;=\;k\times {C_{M}}^{m}\times {C_{N}}^{n}$$

(2) $$k\;=A\times e^{-\frac{E_{a}}{RT}}\;$$

where *v* denotes the reaction rate, *k* the reaction velocity constant at temperature *T*, *C*_M_ and *C*_N_ the initial concentrations of substrates M and N, respectively, *m* and *n* the chemical equilibrium coefficients of substrates M and N, *A* the Arrhenius constant, *e* the base of the natural logarithm, *E*_a_ (J mol^−1^) the experimental activation energy, which can be regarded as a constant independent of temperature, *R* (J mol^−1^ K^−1^) the molar gas constant, and *T* (K) the absolute temperature during the reaction. Thus, increases in the accessible uranium concentration and the environmental temperature can enhance the chemical coordination between uranyl ions and the adsorbent functional groups.^[^ ^15^ ^]^ However, the most feasible adsorbents are usually composed of macrostructures, and the functional groups within the adsorbent are not easily accessible, which reduces the concentration of free uranyl ions around the functional groups inside the adsorbent and further limits the uranium extraction capacity.^[^ ^16^ ^]^ Furthermore, owing to low ion accessibility, the extraction process of uranium by currently available adsorbents usually requires a few weeks or even a few months to reach equilibrium in natural seawater,^[^ ^7a–c^ ^]^ which implies significant operational costs.^[^ ^5a^ ^]^ Thus, it is necessary to develop a new strategy for handling massive amounts of seawater by increasing the accessibility of the functional groups within the adsorbent to uranyl ions.

**Figure 1:**
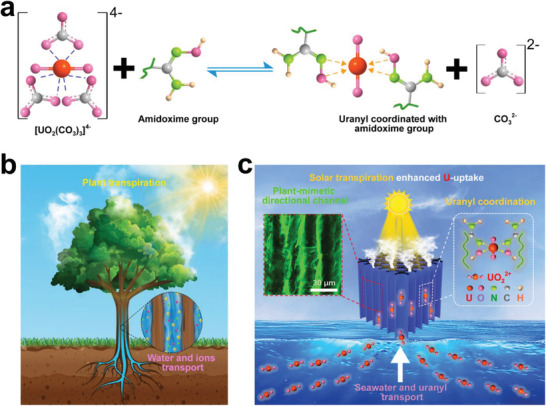
Solar‐transpiration‐enhanced uranium adsorption by plant‐mimetic directional‐channel hydrogel. a) Chemical coordination mechanism between amidoxime group and uranyl ions. b) Schematic of transpiration‐driven water and ion transport in plants. c) Schematic of transpiration‐enhanced uranium extraction from seawater by directional‐channel hydrogel.

Hydrogels are outstanding candidates for uranium recovery from seawater owing to their unique 3D hydrophilic crosslinked networks, which can hold large amounts of water. In contrast to most uranium adsorbents with relatively hydrophobic and dense structures, hydrogels with hydrophilic and loose structures facilitate the easy migration of uranyl ions into their inner structures.^[^ ^17^ ^]^ Recently, hydrophilic hydrogel structures have also been introduced into amidoxime‐functionalized materials to improve their uranium adsorption efficiency.^[^ ^16^ ^]^ However, common hydrophilic 3D crosslinked networks of hydrogels are in the form of natural nanosized mesh structures, which cannot provide satisfactory transport channels for the fast migration of uranyl ions into the adsorbent.^[^ ^18^ ^]^ Herein, inspired by the structures found in plants for water and ion transport, we use composites with suitably oriented hydrophilic macrosize channels to design a new directional‐channel poly(amidoxime) (PAO) hydrogel (DC‐PAO hydrogel) that provides transpiration‐driven seawater transport for enhanced uranium extraction from seawater (**Figure** **1**). In our approach, the upper surface of the DC‐PAO hydrogel was dyed by brushing with 4 wt% melanin aqueous solution, which can be firmly coated to 1.2 ± 0.2 mg cm^−2^ for the highly efficient absorption of sunlight to raise the hydrogel temperature and further increase the transpiration effect to pump seawater. The directional channels of the gel thereby expose more functional sites for uranium adsorption within the channels. Furthermore, the increase in seawater transportation boosts the accessibility of uranium to the functional groups, and the temperature increase together enhances the chemical thermodynamics for the chemical adsorption of uranium. Consequently, the DC‐PAO hydrogel affords a high uranium adsorption speed in natural seawater among all currently available amidoxime group‐based adsorbents.

## 2 Results and Discussion

**Figure 2:**
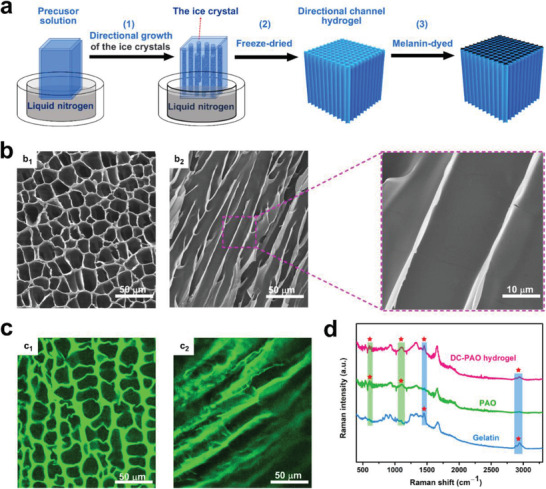
Fabrication and characterization of directional‐channel hydrogel. a) Fabrication process of plant‐mimetic directional‐channel hydrogel. b) SEM images of the (b_1_) cross‐section view and (b_2_) longitudinal view of the dry hydrogel. c) LSCM images of the (c_1_) cross‐section view and (c_2_) vertical‐section view of freshly prepared wet hydrogel. d) Raman spectra of gelatin, PAO, and directional hydrogel.

To fabricate a hydrogel for uranium extraction, we first prepared poly(amidoxime) (Figure S1, Supporting Information)^[^ ^16^ ^]^ and further confirmed its structure by Fourier transform infrared (FTIR) and ^13^C‐NMR analysis. The disappearance of the polyacrylonitrile (PAN) characteristic peak at 2247 cm^−1^ (C≡N stretching) and the appearance of peaks at 1645 cm^−1^ (C═N stretching) and 943 cm^−1^ (N—O stretching) indicate that the nitrile groups in PAN are completely transformed into amidoxime groups (Figure S2, Supporting Information). Meanwhile, the ^13^C‐NMR spectrum shown in Figure S3 (Supporting Information) further confirms the formation of PAO. The uranium adsorption capacity of the hydrogel with different rates of *m*_(gelatin)_/*m*_(PAO)_ (from 10:0 to 3:7) was evaluated (Figure S4, Supporting Information). We note that with the increase in PAO content, the uranium adsorption efficiency gradually decreases. When the *m*_(gelatin)_/*m*_(PAO)_ rate is 5.5:4.5, the uranium adsorption capacity of this hydrogel without channels is the highest, owing to the relatively high PAO content and high uranium adsorption efficiency of PAO. Next, different plant‐mimetic directional‐channel hydrogels with the same proportion of *m*_(gelatin)_/*m*_(PAO)_ (5.5:4.5) were fabricated based on the technology of ice‐crystal growth to form an oriented‐channel structure (**Figure** **2**a). Upon immersing the bottom of container with the 70 °C precursor solution in −197 °C liquid nitrogen, long ice crystals in the precursor solution form in the bottom‐up direction because heat is conducted from the top of the container to the bottom.^[^ ^19^ ^]^ After drying in a lyophilizer, a directional‐channel structure is formed in the biomimetic hydrogel (Figure S5, Supporting Information). The diameter of the channel can be adjusted from 10 ± 5 to 45 ± 5 µm by changing the solid contents (including the gelatin and PAO content) of the precursor solution from 7% to 12% (Figure S6, Supporting Information). Hydrogels with different directional‐channel structures exhibit different uranium adsorption capacities and solar evaporation efficiencies. In the study, the hydrogel fabricated with 9% solid content, with a channel diameter of 20 ± 5 µm (Figure 2b) with high porosity (Figure S6, Supporting Information), was chosen for subsequent investigations because of its high uranium adsorption capacity (Figure S7, Supporting Information) and high solar evaporation efficiency (Figure S8, Supporting Information). Laser scanning confocal microscope (LSCM) images of the DC‐PAO hydrogel revealed that the directional channels were maintained satisfactorily in seawater, i.e., the channel size in seawater is close to that of the dry state owing to the channel's firmly porous structure (Figure S9, Supporting Information); thus, this structure can aid the transport of seawater during practical application (Figure 2c). Additionally, the Raman spectra indicate that the PAO is dispersed and fixed in this directional‐channel hydrogel, thereby indicating the successful fabrication of the material (Figure 2d). Our hydrophilicity analysis revealed that the hydrogel cross section exhibits high hydrophilicity, which again can benefit the transport of seawater from the hydrogel bottom to the top (Figure S10, Supporting Information).

**Figure 3:**
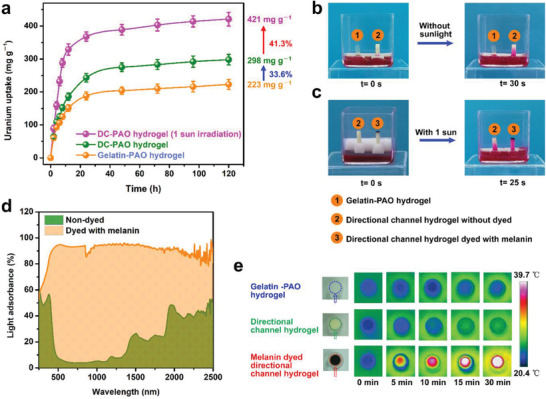
Uranium adsorption performance in simulated seawater and mechanism for enhanced uranium adsorption. a) Uranium adsorption capacity of the channel‐less hydrogel, directional‐channel hydrogel under darkness, and directional‐channel hydrogel under 1 sun irradiation, in 8 ppm uranium‐spiked seawater (*n* = 3). Data are shown as means ± SD. b) Seawater transport ability of channel‐less hydrogel and directional‐channel hydrogel without sunlight irradiation. c) Seawater transport ability of directional‐channel hydrogel dyed with and without melanin under sunlight irradiation. d) Light absorbance performance of directional‐channel hydrogel dyed with and without melanin. e) Temperature distributions of hydrogel specimens immersed in water under 1 sun irradiation.

The uranium adsorption capacity of the hydrogel was determined in uranium‐spiked seawater with a uranium concentration of 8 ppm. Compared with the traditional gelatin‐PAO hydrogel with the same chemical components but no directional‐channel structure, the uranium adsorption capacity of the directional‐channel hydrogel increases significantly by 33.6% from 223 to 298 mg g^−1^ in 8 ppm uranium‐spiked seawater within 120 h (**Figure** **3**a). The observed enhancement of the uranium adsorption capacity is attributed to the presence of the directional channels for seawater and ion transport and the exposure of more functional groups in the channels for uranium binding. Owing to the introduction of the directional channels in the hydrogel, with the use of rhodamine B as an indicator of seawater transportation, we find that the seawater transportation speed increases by 6.65 times from 0.045 to 0.299 mm s^−1^ at 30 °C without sunlight (Figure 3b). When black melanin dye is applied to the upper surface of the hydrogel, the seawater transport speed further increases by 1.59 times from 0.306 to 0.487 mm s^−1^ under 1 sun irradiation (Figure 3c). This directional‐channel hydrogel dyed with melanin exhibits high absorption over a broad wavelength range of sunlight radiation with 90.5% energy‐adsorption efficiency, especially in the infrared region (Figure 3d). However, the directional‐channel hydrogel without melanin dye only exhibits an adsorption of 23.5% when exposed to the light. When compared with the cases of the bare gelatin‐PAO hydrogel with no channels and non‐dyed directional hydrogels, which scarcely absorb solar energy, solar irradiation can rapidly increase the temperature of the melanin‐dyed black hydrogel (Figure 3e). Under the light intensity of 1 sun, the temperature of the dyed hydrogel increases from 20.0 to 32.8 and 37.3 °C within 5 and 10 min, respectively, and thereafter maintains a temperature of >38.6 °C after irradiation for 15 min. Owing to the high solar energy absorption ability resulting in a significant temperature increase and the role of the plant‐mimic directional‐channel hydrogel in transporting seawater, the hydrogel affords an ultra‐efficient solar desalination performance of 2.86 kg m^−2^ h^−1^ under 1 sun intensity (Figure S8 and Table S1, Supporting Information). In addition, we note that the desalinated water only contains extremely low concentrations of four major metal elements, including Na (0.211 mg L^−1^), K (0.014 mg L^−1^), Ca (0.015 mg L^−1^), and Mg (0.024 mg L^−1^) (Figure S11, Supporting Information). The dye applied to the hydrogel causes a temperature increase under solar irradiation, and the adsorption chemical thermodynamics for uranium adsorption are further enhanced with this increase in temperature. Compared with the hydrogel without both directional channels and solar illumination, the equilibrium uranium adsorption capacity of the directional‐channel hydrogel increases by 88.7% from 223 to 421 mg g^–1^ in 8 ppm uranium‐spiked seawater under 1 sun intensity (Figure 3a).

Similar to the other amidoxime group‐based adsorbents, this plant‐mimetic directional‐channel hydrogel also exhibits high selectivity for uranium, but not the other completion metal ions in seawater (Figure S12 and Table S2, Supporting Information). A survey of the X‐ray photoelectron spectroscopy (XPS) spectra shows that the characteristic double peaks of U4f in uranyl ions are detected in the hydrogel after uranium adsorption, which confirms the binding of uranium (Figure S13, Supporting Information). Furthermore, the uranium adsorption of the hydrogel can be confirmed by the EDS‐mapping images (Figure S14, Supporting Information) and the high‐resolution XPS spectra of U4f, which show that uranium maintains its initial valence in the uranyl ion after adsorption (Figure S15, Supporting Information). After uranium adsorption, the hydrogel maintains an integrated channel structure, which implies the reusability of the adsorbent (Figure S16, Supporting Information). Our analysis of the hydrogel recyclability shows that the bound uranium can be efficiently eluted by an elution solution containing 1.0 m Na_2_CO_3_ and 0.1 m H_2_O_2_ within 20 min (Figure S17, Supporting Information). After reuse for ten adsorption–desorption cycles, the hydrogel still maintains 79.3% of the initial uranium adsorption capacity, indicating an average 2.3% loss of adsorption capacity after each reuse cycle (Figure S18, Supporting Information). The elution efficiency decreases only slightly from 92.6% to 85.4% after the hydrogel is reused for ten cycles. These results prove that this plant‐mimetic directional‐channel hydrogel affords outstanding reusability.

**Figure 4:**
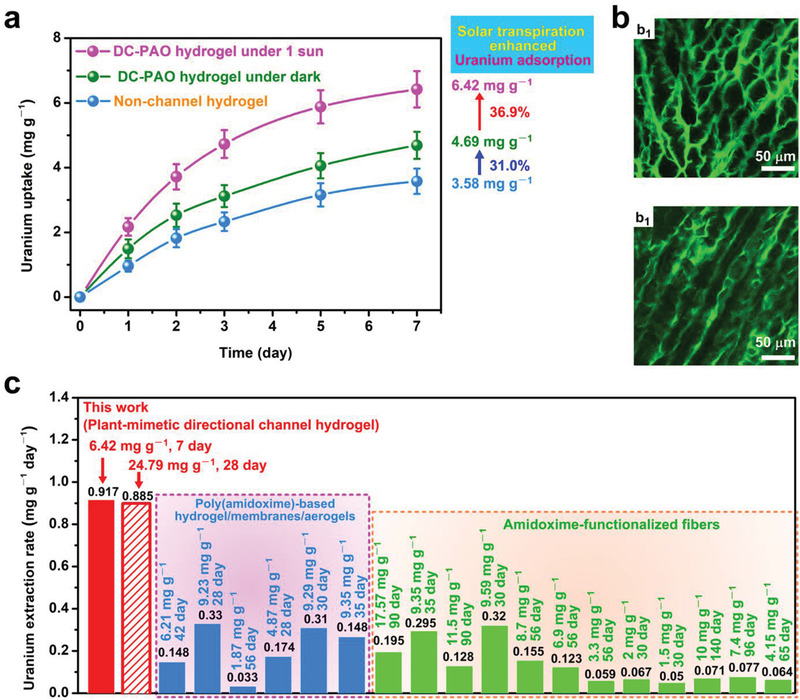
Uranium extraction from natural seawater. a) Uranium extraction performance in natural seawater (*n* = 3). Data are shown as means ± SD. b) Images of the (b_1_) cross‐section view and (b_2_) longitudinal view of the used wet hydrogel. c) Comparison of the uranium adsorption rates among existing adsorbents applied to natural seawater. The extrapolated cumulative uranium extraction capacity based on adsorbent use for four cycles is indicated by the “slashed” column. The corresponding adsorbents are listed in Table S3 (Supporting Information).

To further determine the uranium recovery ability of the directional‐channel hydrogel in natural seawater without further uranium addition, coastal seawater collected from Wanning city in Hainan Province, China, was used for further experiments. Under 1 sun irradiation, the DC‐PAO hydrogel exhibits a high uranium extraction capacity of 6.42 ± 0.56 mg g^−1^ after being tested for 7 d, which is higher than those of the DC‐PAO hydrogel not exposed to irradiation (4.69 ± 0.42 mg g^−1^) and the channel‐less hydrogel (3.58 ± 0.39 mg g^−1^) by 36.89% and 79.33%, respectively (**Figure** **4**a; Figure S19, Supporting Information). The LSCM images show that the hydrogel maintains the plant‐mimetic directional‐channel structure satisfactorily, and thus, it can continuously provide suitable channels for the transportation of water and uranyl ions for a relatively long time (Figure 4b). Compared with existing uranium adsorbents, this transpiration‐enhanced plant‐mimetic directional‐channel hydrogel affords an outstanding uranium adsorption rate of 0.917 mg g^−1^ d^−1^ (Figure 4c). Based on the average capacity loss after each reuse process, after reusing the DC‐PAO hydrogel for four cycles, we extrapolated the hydrogel capacity to estimate a cumulative uranium extraction capacity of 24.79 mg g^−1^ within 28 d with an adsorption rate of 0.885 mg g^−1^ d^−1^. To the best of our knowledge, this is the fastest uranium adsorption rate among currently available amidoxime group‐based adsorbents in natural seawater (Figure 3c; Table S3, Supporting Information). This result indicates that the design of plant‐mimetic directional channels is a promising strategy for boosting the speed of uranium extraction from seawater.

## 3 Conclusion

In conclusion, we proposed a new method for fabricating a plant‐mimetic directional‐channel gelatin‐poly(amidoxime) hydrogel via a simple directional‐freezing technique. The fabricated directional‐channel hydrogel affords a high transpiration‐enhanced uranium adsorption performance together with an ultra‐efficient solar desalination performance of 2.86 kg m^−2^ h^−1^. Among the most state‐of‐the‐art amidoxime‐group‐based adsorbents for uranium extraction from seawater, this plant‐mimetic transpiration‐enhanced hydrogel affords the fastest uranium adsorption rate of 0.917 mg g^−1^ d^−1^, owing to hydrophilic directional channels that transport seawater together with uranyl ions and the increase in temperature (due to sunlight absorption) that results in accelerated chemical thermodynamics for the chemical adsorption of uranium. Compared with the hydrogel without plant‐mimetic transpiration, the uranium extraction capacity of our plant‐mimetic directional‐channel hydrogel increased by 79.33% to 6.42 mg g^−1^ within 7 d of testing; this corresponds to a shortened extraction time by a factor of at least four when compared with other amidoxime‐group based adsorbents with similar uranium extraction capacities from natural seawater. In summary, our bio‐inspired low‐cost plant‐mimetic directional‐channel hydrogel prototype efficiently enhances uranium extraction from seawater and also affords highly efficient solar desalination. We believe that our approach will also inspire the comprehensive utilization of other resources in seawater using similar synergistic strategies.

## Conflict of Interest

The authors declare no conflict of interest.

## Supporting information

Supporting Information

## Data Availability

Research data are not shared.
