# Supplementary material for: Accelerated Chemical Thermodynamics of Uranium Extraction from Seawater by Plant‐Mimetic Transpiration
Source: Adv Sci (Weinh). 2021 Oct 28;8(24):2102250. doi: 10.1002/advs.202102250 (PMC8693040; doi:10.1002/advs.202102250)
Supplement: Supplementary file 1 — Supporting Information [file ADVS-8-2102250-s001.pdf]

© 2021 Wiley-VCH GmbH

ADVANCED SCIENCE
Open Access

Supporting Information

for *Adv. Sci.*, DOI: 10.1002/advs.202102250

Accelerated Chemical Thermodynamics of Uranium Extraction from Seawater by Plant-Mimetic Transpiration

Ning Wang, Xuemei Zhao, Jiawen Wang, Bingjie Yan, Shunxi Wen, Jiacheng Zhang, Ke Lin, Hui Wang, Tao Liu, Zhenzhong Liu, Chunxin Ma, Jianbao Li, and Yihui Yuan**

Supporting Information

Accelerated Chemical Thermodynamics of Uranium Extraction from Seawater by Plant-Mimetic Transpiration

Ning Wang, Xuemei Zhao, Jiawen Wang, Bingjie Yan, Shunxi Wen, Jiacheng Zhang, Ke Lin, Hui Wang, Tao Liu, Zhenzhong Liu, Chunxin Ma, Jianbao Li, and Yihui Yuan**

Prof. N. Wang, X. Zhao, J. Wang, B. Yan, S. Wen, J. Zhang, K. Lin, Dr. H. Wang, Prof. T. Liu, Prof. C. Ma, Prof. J. Li, Prof. Y. Yuan
State Key Laboratory of Marine Resource Utilization in South China Sea, Hainan University, Haikou 570228, P. R. China
E-mail: yyhheda@126.com (Y. Yuan), machunxin@hainanu.edu.cn (C. Ma)

Prof. C. Ma, Dr. Z. Liu

Research Institute of Zhejiang University-Taizhou, Taizhou 318000, P. R. China

Experimental Section

Materials: Polyacrylonitrile (PAN, 99%), hydroxylamine hydrochloride ($NH_2OH{\cdot}HCl$, 99%), sodium hydroxide (NaOH, 99.5%), N, N-Dimethy-lformamide (DMF, 99.9%), arsenazo (III) (95%), vanadium sulfate hydrate ($VOSO_4{\cdot}xH_2O$, 99.9%), sodium carbonate (Na_2CO_3, 99.8%) were phrased from Macklin. Potassium chloride (KCl,99.5%), sodium chloride (NaCl, 99.5%), calcium chloride ($CaCl_2$, 96%), nickel chloride hexahydrate ($NiCl_2{\cdot}6H_2O$, 98%), magnesium Chloride (96%), ferric chloride hexahydrate ($FeCl_3{\cdot}6H_2O$, 99%), copper sulfate pentahydrate ($CuSO_4{\cdot}5H_2O$, 99%), C_2H_5OH (99.5%), hydrochloric acid (12 mol/L) and acid black 2 (water-soluble melanin, $C_{22}H_{14}N_6O_9S_{2\text{-}3.2}Na$, 99%) were phrased from Xilong Scientific Co. Ltd. Cobalt uranium hexahydrate nitrate [$UO_2(NO_3)_2{\cdot}6H_2O$, 99%]. chloride hexahydrate ($CoCl_2{\cdot}6H_2O$, 99%), was phrased from Chushengwei Chemical Co., Ltd. All the chemicals above were used as received without further purification. All the natural seawater was collected from the Qiongzhou strait nearby the Haikou city of Hainan province in China and was used after filtrated with a 0.45 μm filter.

Characterization: FT-IR spectra were observed on a Perkin-Elmer LR-64912C (FT-IR, LR 64912C, Perkin-Elmer, USA). UV-Vis absorption spectra were conducted with a spectrophotometer (UV1800PC, AuCy Instrument, China). Elemental electron binding energy were obtained on an X-ray photoelectron spectroscopy (XPS, Thermo escalab 250Xi Thermo, USA). Positional state of carbon studies was detected on Nuclear magnetic resonance (NMR, Bruker AVANCE III 600M, Bruker, Germany). The hydrophilic property of the Wood-mimetic directional channel hydrogel was observed on a contact angle measuring instrument (JC2000D, Shanghai Zhongchen digital technic apparatus CO., LTD, China). The microscopic morphology and structure of the hydrogels in dry state were obtained on a field emission scanning electron microscope (SEM, S-4800, HITACHI, Japan). The microscopic morphology and structure of the hydrogels in seawater, which (dry gels) were cut to 30-50 μm thickness and dyed by

fluorescent agent seawater solution, were observed through a laser scanning confocal microscope (LSCM, Deiss, Germany). The pH values were detected via a pH meter (F2, Mettler Toledo, Germany). Adsorption selectivity of the directional-channel hydrogel on the uranyl ion and other metal ions in a simulated seawater was studied on inductively coupled plasma mass spectrometer (ICP-OES: ICPOES730; ICP-MS: Agilent 7500ce: Agilent, USA). The Uranium(VI) concentration of the natural seawater was measured through the other ICP-MS (Thermoscientific iCAP RQ, Germany).

Synthesis of the Poly(amidoxime) (PAO): The poly(amidoxime) (PAO) was synthesized according to the published literature (**Figure S1a**).[1] $NH_2OH{\cdot}HCl$ (4.17 g, 60.0 mmol) was dissolved in DMF (45.0 mL) in a round-bottom flask heated by a water bath at 45 °C. Na_2CO_3 (2.87 g, 27.0 mmol) and NaOH (0.72 g, 18.0 mmol) were then added slowly. After stirring with a magnetic stirring apparatus for 3 h, PAN (3.18 g, 60.0 mmol) was added and dissolved completely for at least 30 min, then reacted at 65 °C for 24 h. Finally, Na_2CO_3 (1.43 g, 13.5 mmol) and NaOH (0.36 g, 9.0 mol) were replenished successively, and continuously reacted at 65 °C for 24 h. The reaction mixture was centrifuged, and then the supernatant was dropped into 500 mL of ultra-pure water to precipitate a white floc. After filtering and gathering, the precipitate was dried in a vacuum at 55 °C for 12 h to obtain the as-prepared PAO, which can be dissolved with 0.15 mol/L NaOH (**Figure S1b**).

Calculation of the Uranium Adsorption Capacity: The uranium concentrations in U-spiked water and U-spiked seawater were measured using their UV-Vis absorption spectra, based on arsenazo (III) method, which can coordinate with the uranyl ion and exhibits an intense specific peak at 652 nm, according to the literature.[2] Curvilinear regressions of the uranium concentration-absorbance in ultrapure-water and seawater solution were achieved. The uranium concentrations can be calculated using the two standard curves.

The hydrogel containing 10 mg of dry gel was immersed in a 2.0 L U-spiked ultrapure-water or U-spiked seawater solutions and stirred with a table concentrator, the uranium adsorption mass of the hydrogel-based adsorbent can be calculated based on formula (1):

$$M_U = (C_o - C_t) \times V \quad (1)$$

where M_U is the uranium adsorption mass of the hydrogel, and C_o and C_t are the uranium concentrations of the U-spiked aqueous solution at different times. V is the volume of the U-spiked solution.

The uranium adsorption capacity of the hydrogel can be calculated uniformly according to formula (2):

$$Q_{hydrogel} = M_U/M_{dry\ gel} \quad (2)$$

where $Q_{hydrogel}$ is the quantity of uranium the hydrogel can adsorb, M_U is the uranium adsorption mass of the hydrogel, and $M_{dry\ gel}$ is the dry mass of the hydrogel.

The uranium adsorption capacity of the PAO in hydrogel can be calculated uniformly according to formula (3):

$$Q_{PAO} = M_U/M_{PAO} \quad (2)$$

where Q_{PAO} is the quantity of uranium the hydrogel can adsorb, M_U is the uranium adsorption mass of the hydrogel, and M_{PAO} is the mass of the PAO in hydrogel.

Method of the Uranium Adsorption-Desorption Cycle of the hydrogel: This DC-PAO hydrogel with 10 mg dry mass was immersed in 8 ppm U-spiked seawater (2.0 L) for 12 h to measure its uranium adsorption capacity. According to the literature,[3] 11.7 mL of 30 wt% H_2O_2 aqueous solution and 106 g Na_2CO_3 were dissolved in ultrapure water to prepare the 1000 mL elution solution. The uranium-loaded hydrogel was immersed in the eluent (200 mL). After stirring for 40 min, the adsorbed uranium in the hydrogel can be desorbed. We can regenerate the DC-PAO hydrogel simply by immersing it in 500 mL pure water and replacing the water

for three times. The regenerated hydrogel was utilized for the subsequent adsorption-desorption cycles.

Method of the Solar transpiration and Uranium Extraction from the Natural Seawater: The directional channel hydrogel sample (30 mg) with melanin-dyed upper surface was immobilized in a cylindrical cavity with 2.0 cm diameter of a white plastic foam **(Figure S16a)**. Then, this system was anchored on the surface of 100 kg natural seawater in a bucket (**Figure S1b**) and extracted uranium from nature seawater for 7 days under the continuous irradiation of 1 sun density via a sunlight-simulated xenon lamp. Two contrast tests of the non-channel hydrogel and the directional channel hydrogel were also conducted by the same method described above without irradiating by sunlight. Every 24 h, 50 mL of seawater samples were collected and the uranium adsorption capacity of the hydrogel was evaluated by the ICP-MS.

Supporting Figures

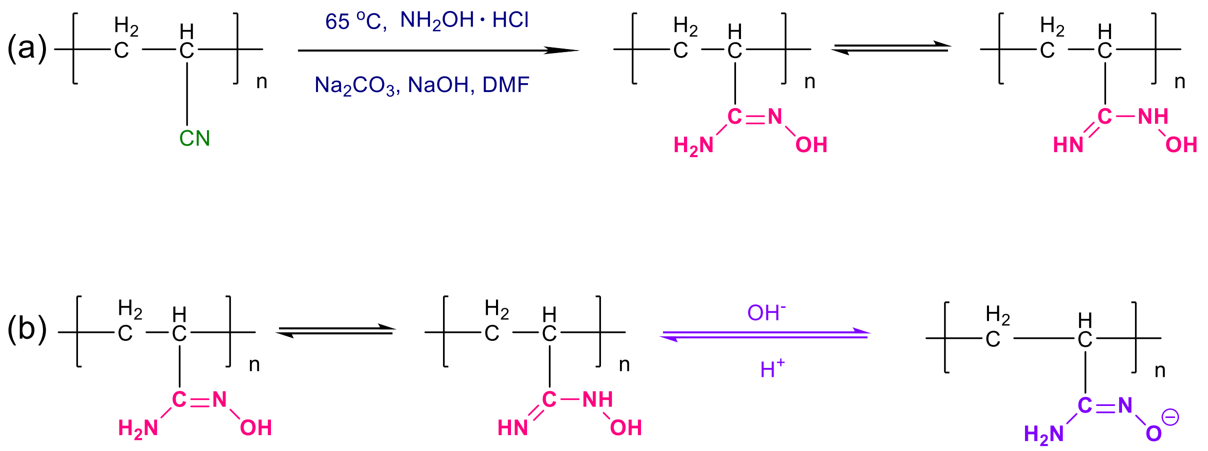

Figure S1. (a) Synthesis of neutral PAO; (b) Preparation of water-soluble PAO containing negative charges.

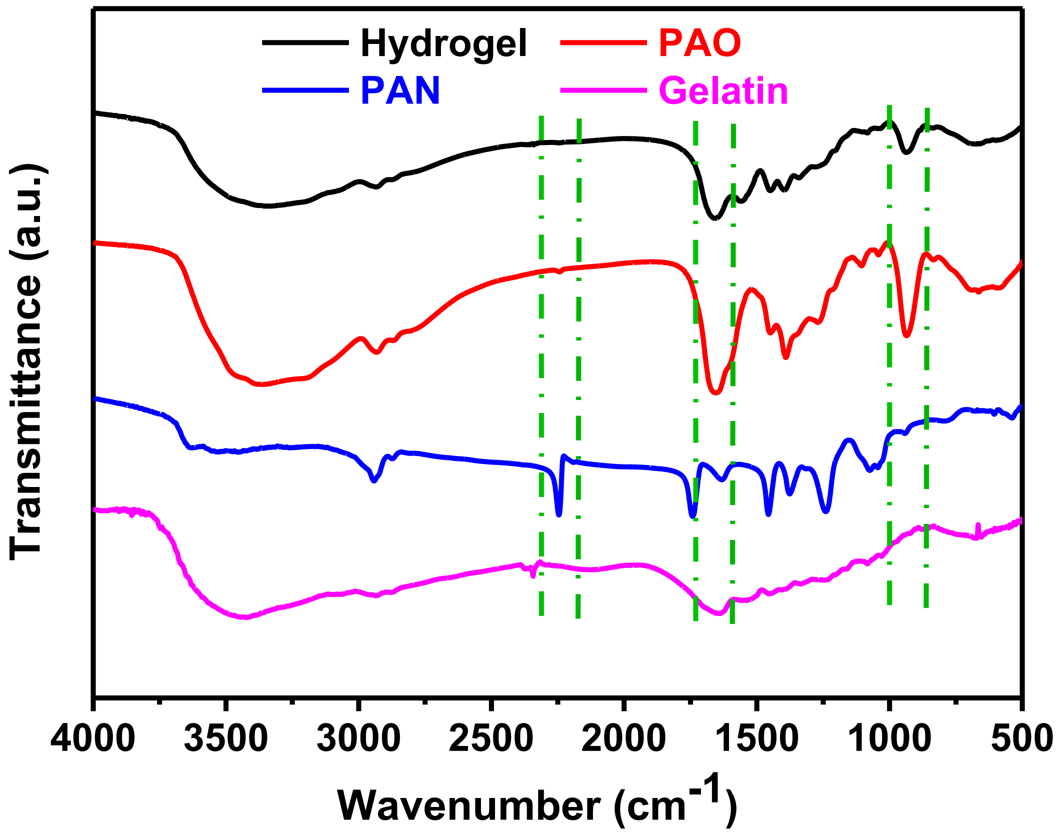

Figure S2. FT-IR spectra of the gelatin, PAN, PAO and the directional macro-porous hydrogel, respectively

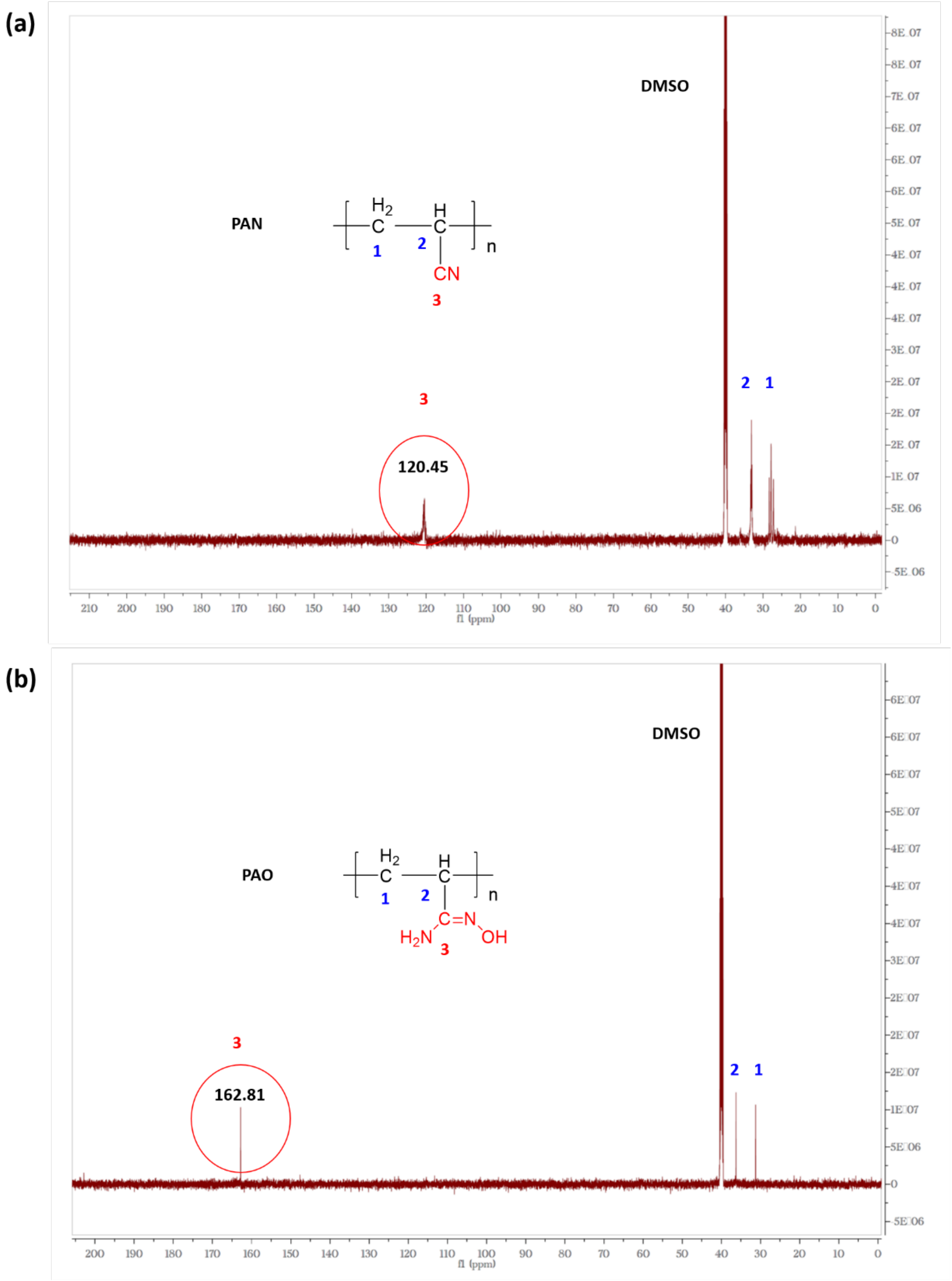

Figure S3. The ^{13}C-NMR spectra of (a) PAN and (b) PAO in DMSO-D_6, respectively.

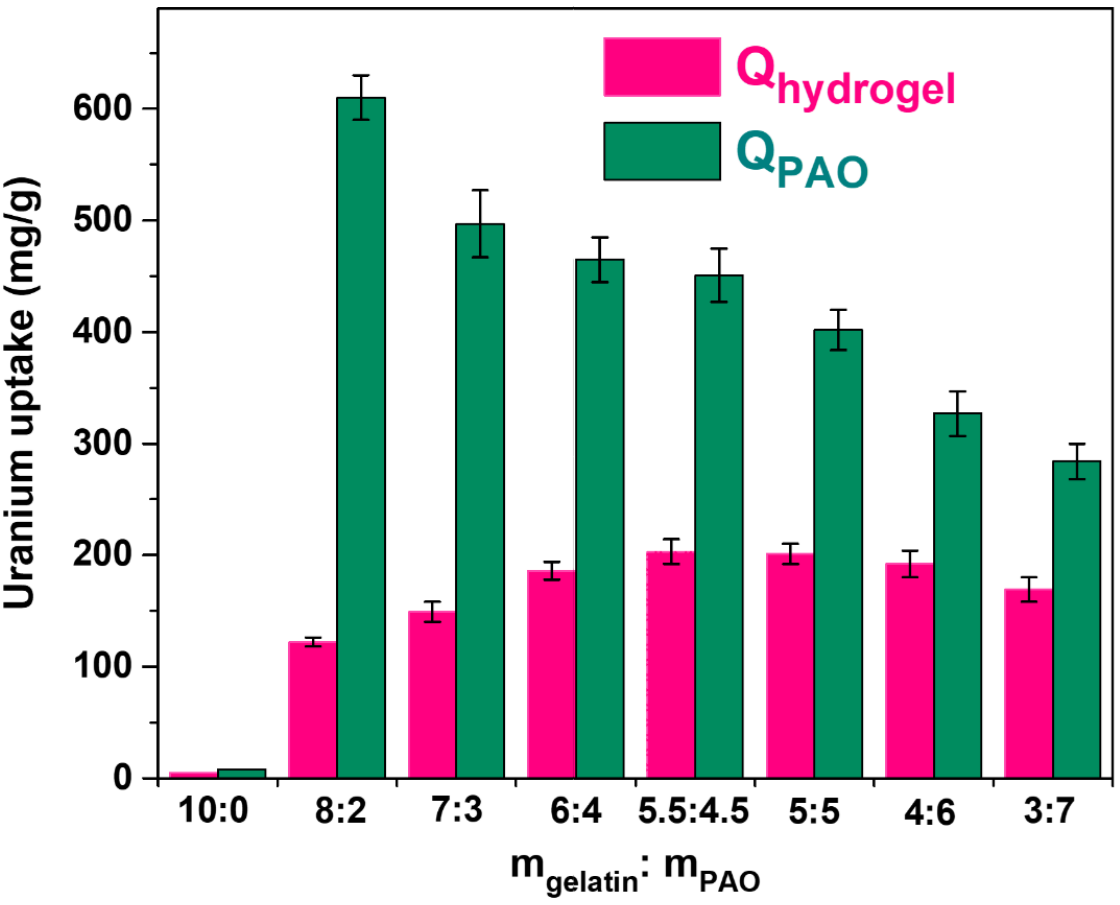

Figure S4. The uranium adsorption capacity of hydrogel ($Q_{hydrogel}$) and PAO (Q_{PAO}) with different mass ratio of gelatin/PAO and constant 10 wt% of total dry matter after immersed in 8 ppm uranium-spiked seawater for 48 h (n = 3). Data are shown ss means ± SD.

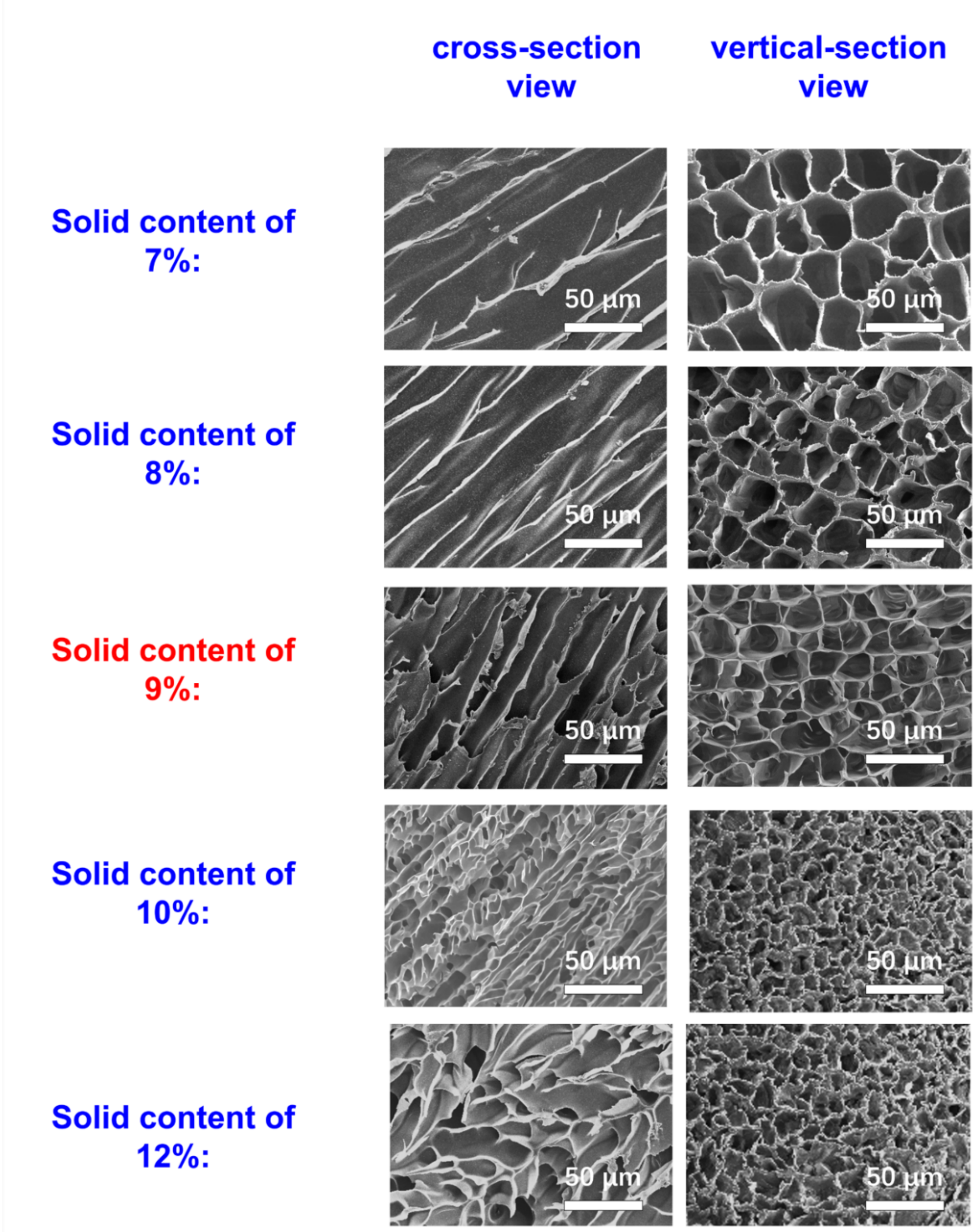

Figure S5. SEM images of plant-mimetic directional channel hydrogels with different solid concentration of the hydrogel precursor solution.

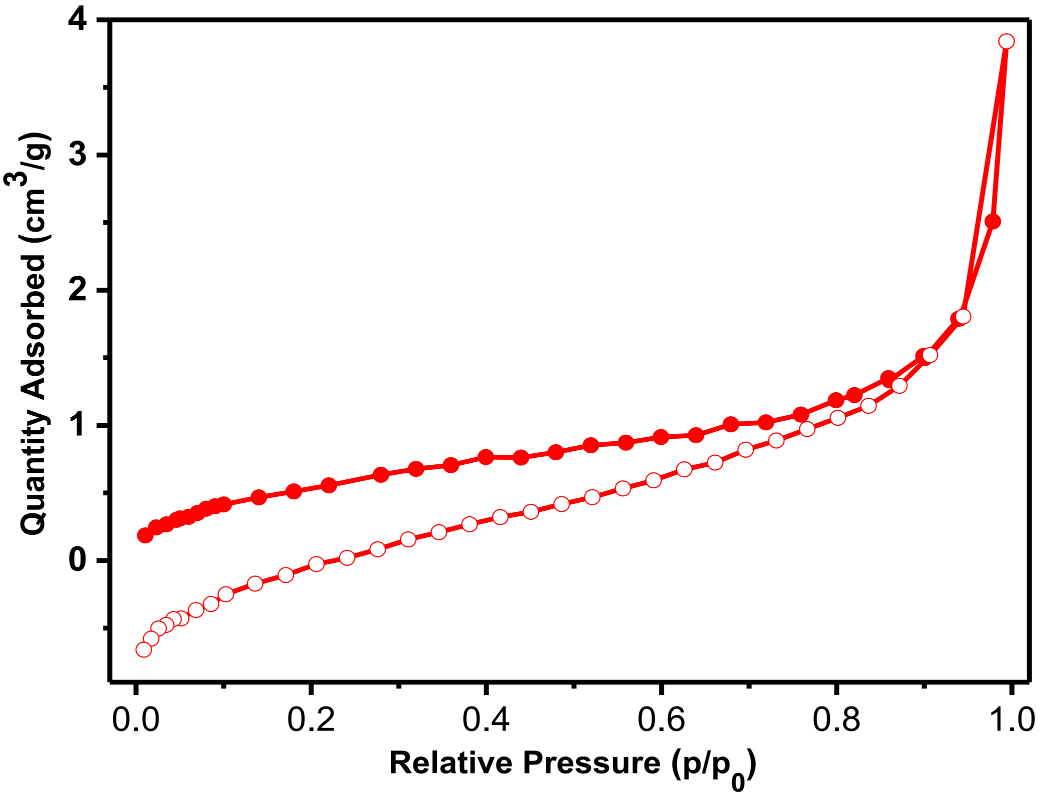

Figure S6. N_2 adsorption-desorption isotherms of the dry hydrogel by BET at 77 K.

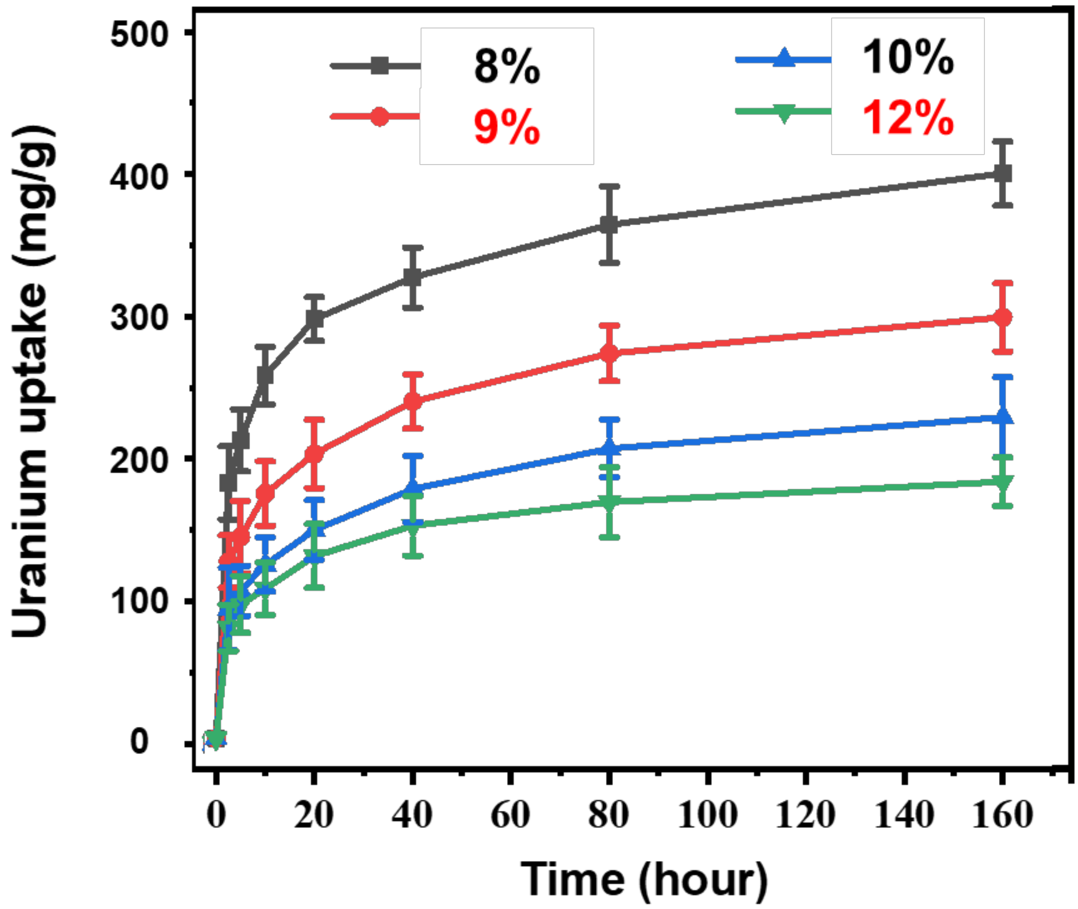

Figure S7. Comparison of the uranium adsorption capacity of directional channel hydrogels fabricated by different solid content in 8 ppm U-spiked seawater (*n* = 3). Data are shown ss means ± SD.

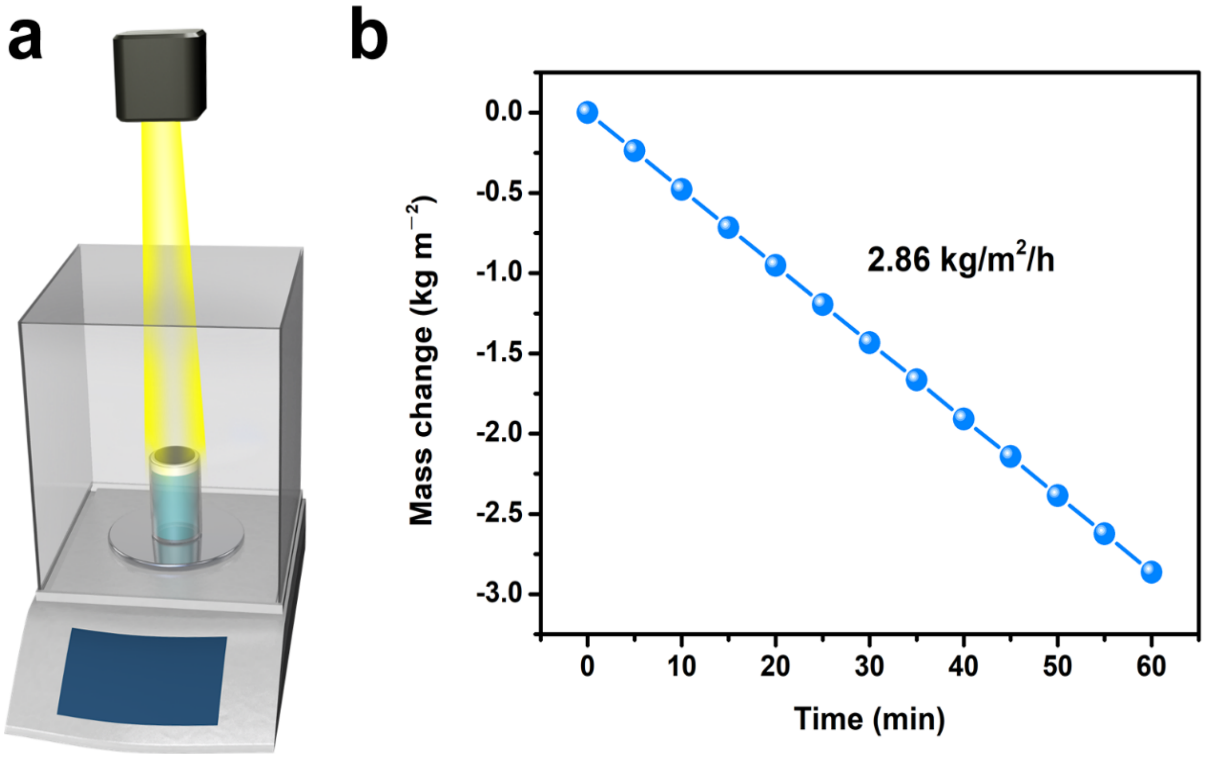

Figure S8. Solar desalination performance of the DC-PAO hdyrogel with 9wt% dry mass in uranium spiked seawater. (a) Equipment used for testing the solar desalination ability of the DC-PAO hydrogel. (b) The solar evaporation efficiency of the melanin dyed DC-PAO hydrogel under 1 sun.

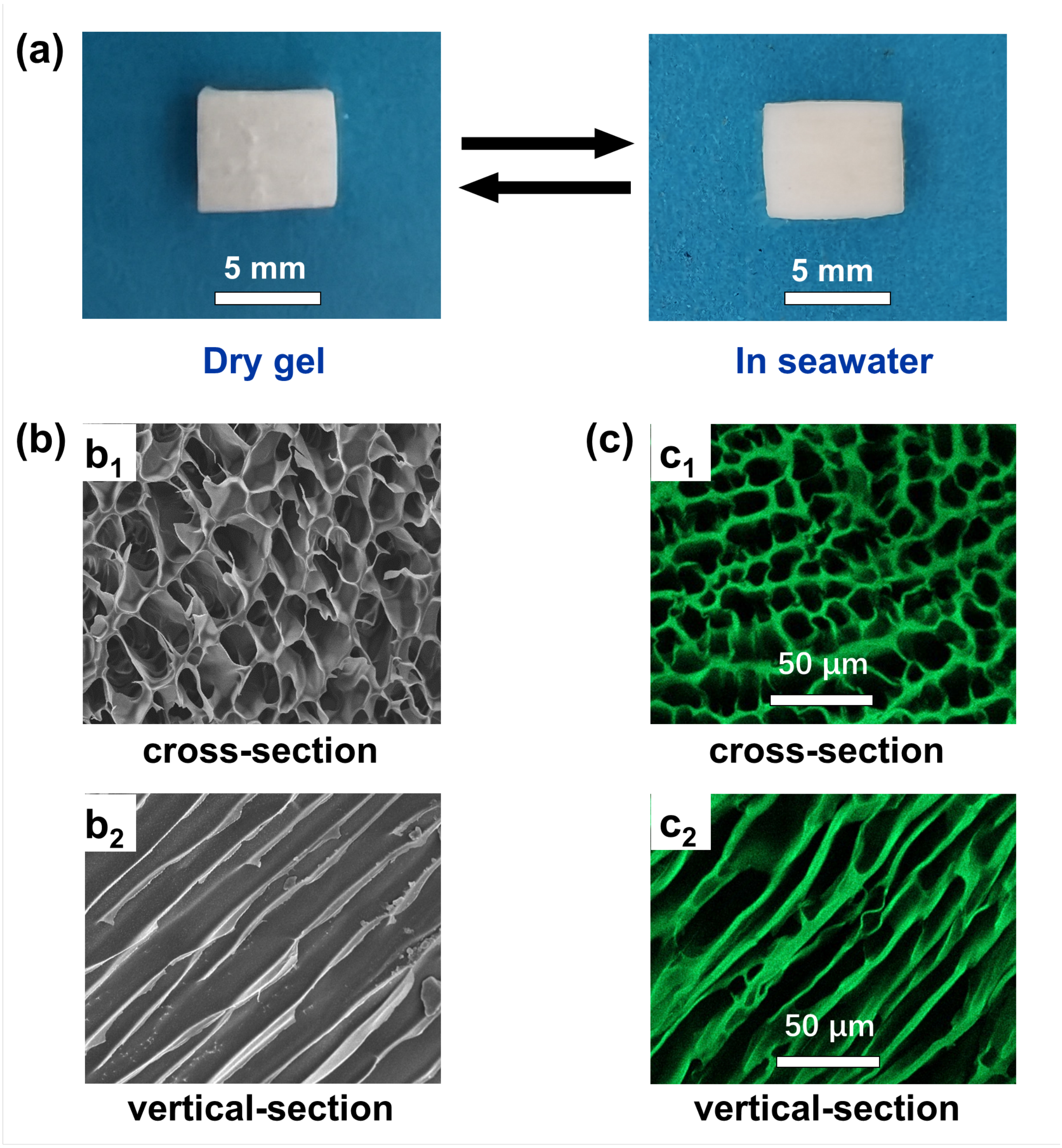

Figure S9. Comparison of the (a) macro size and (b-c) micro morphology by SEM and LSCM images between the DC-PAO hydrogel in dry state and in seawater respectively.

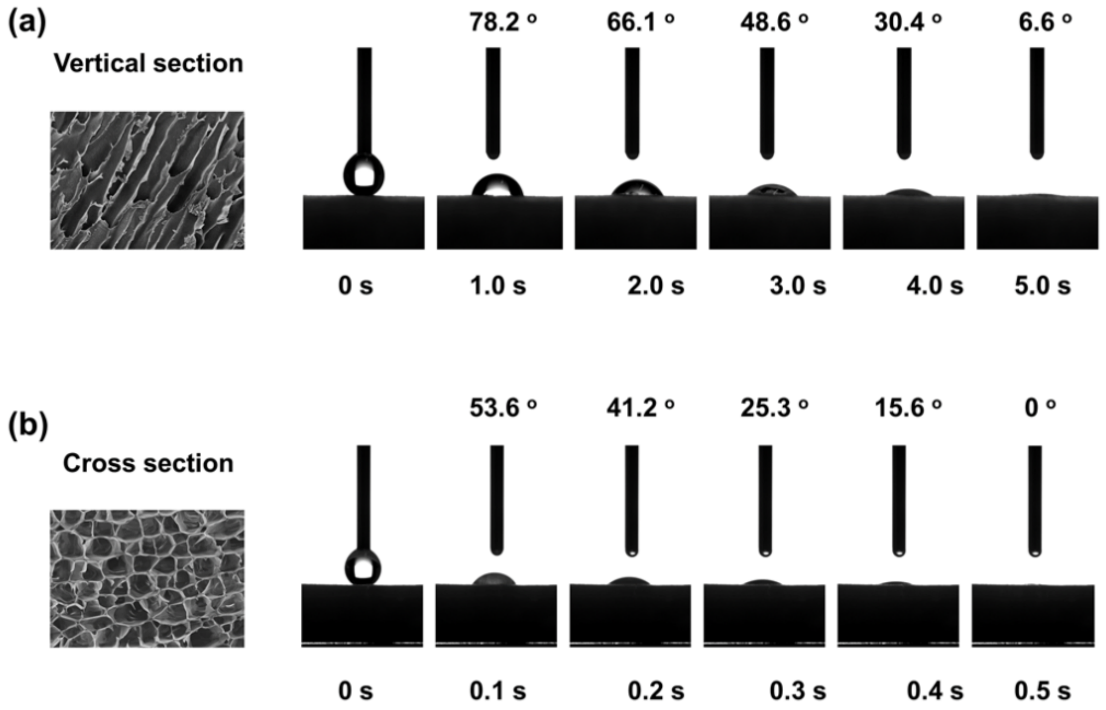

Figure S10. Hydrophilicity of the (a) vertical section and (b) cross section of the directional channel hydrogel.

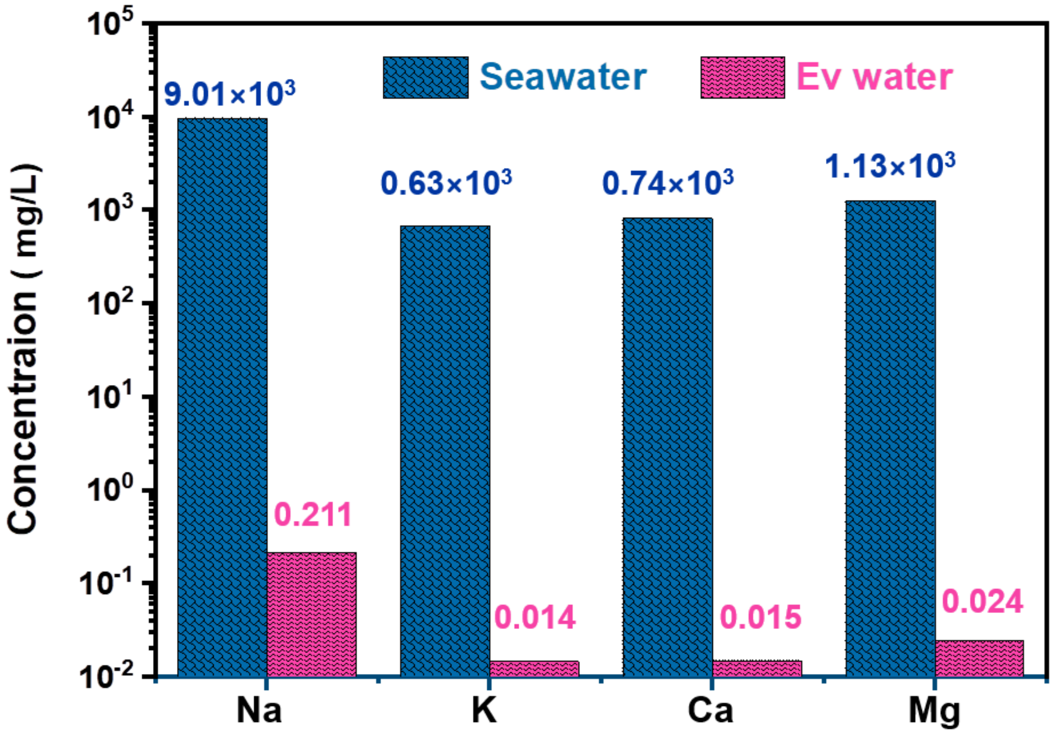

Figure S11. The concentration of major metallic elements in the desalinated water by this plant-mimetic transportion method with the DC-PAO hydrogel.

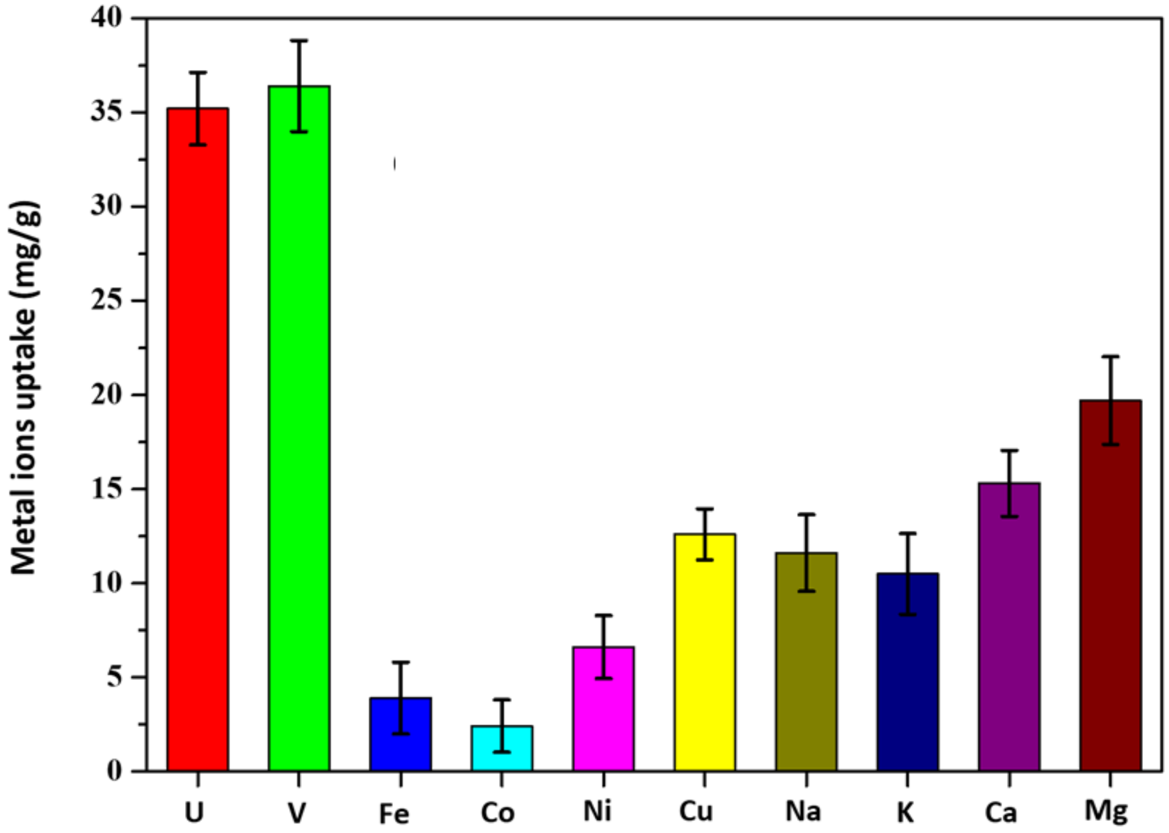

Figure S12. Adsorption selectivity of the plant-mimetic directional channel hydrogel in a modified seawater for 48 h (*n* = 3). Data are shown ss means ± SD. U, V, Fe, Co and Ni and Cu were added into the seawater up to as 100 times as that of the seawater respectively. Na, Ca, Mg, and K were not added and were equal to their concentration in seawater.)

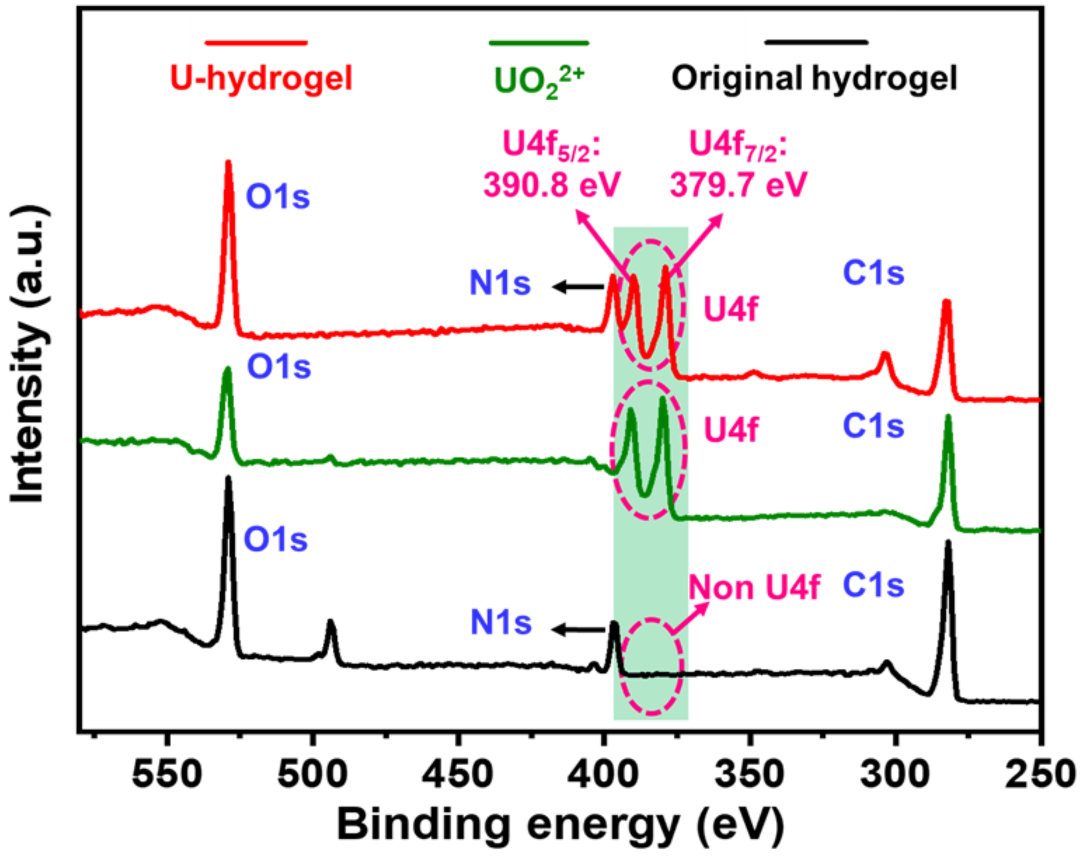

Figure S13. XPS spectra of the U-loaded DC-PAO hydrogel and the original DC-PAO hydrogel.

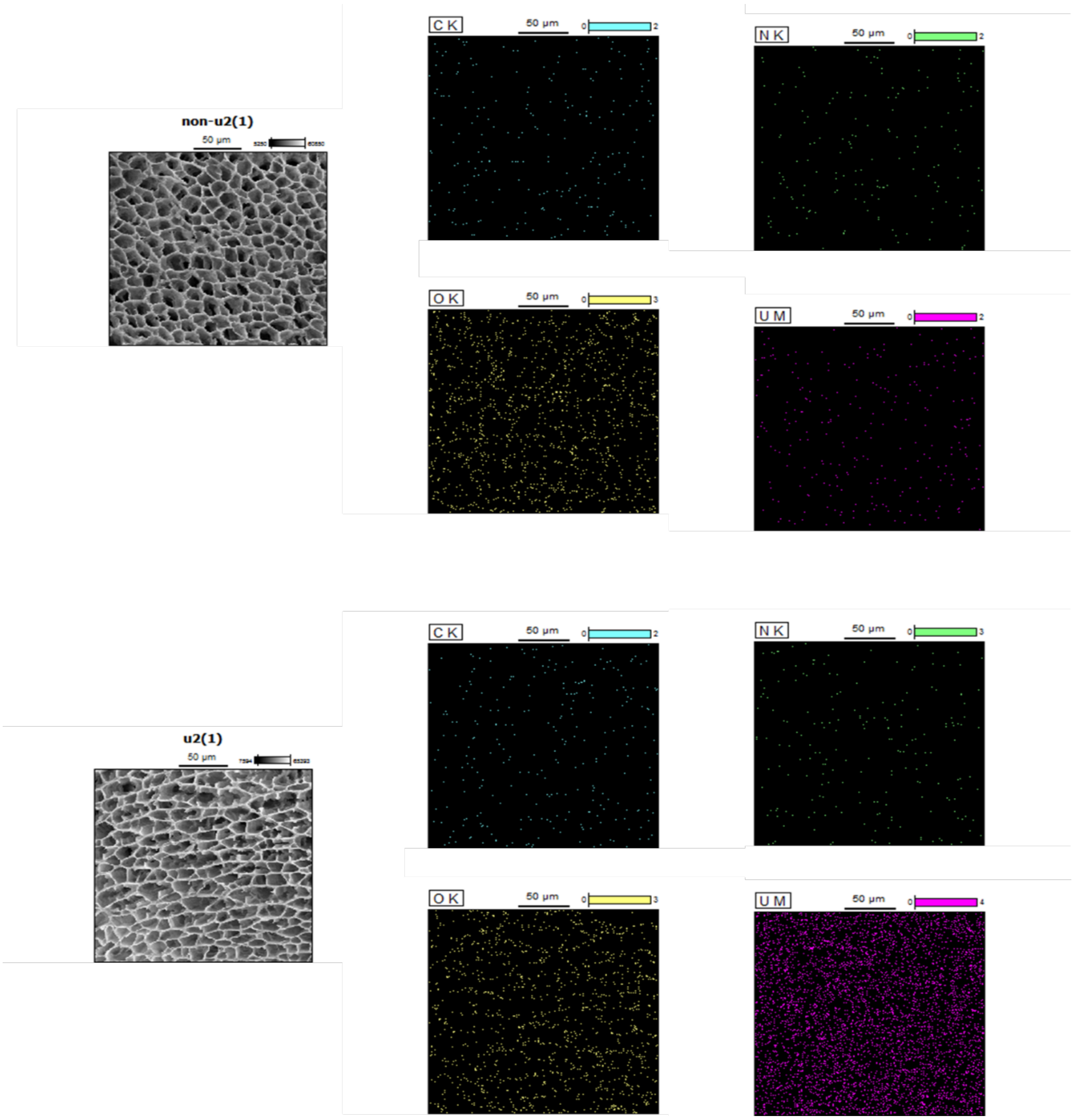

Figure S14. Comparison of the C, O, N and U in EDS mappings between the original DC-PAO hydrogel and the uranium loaded DC-PAO hydrogel.

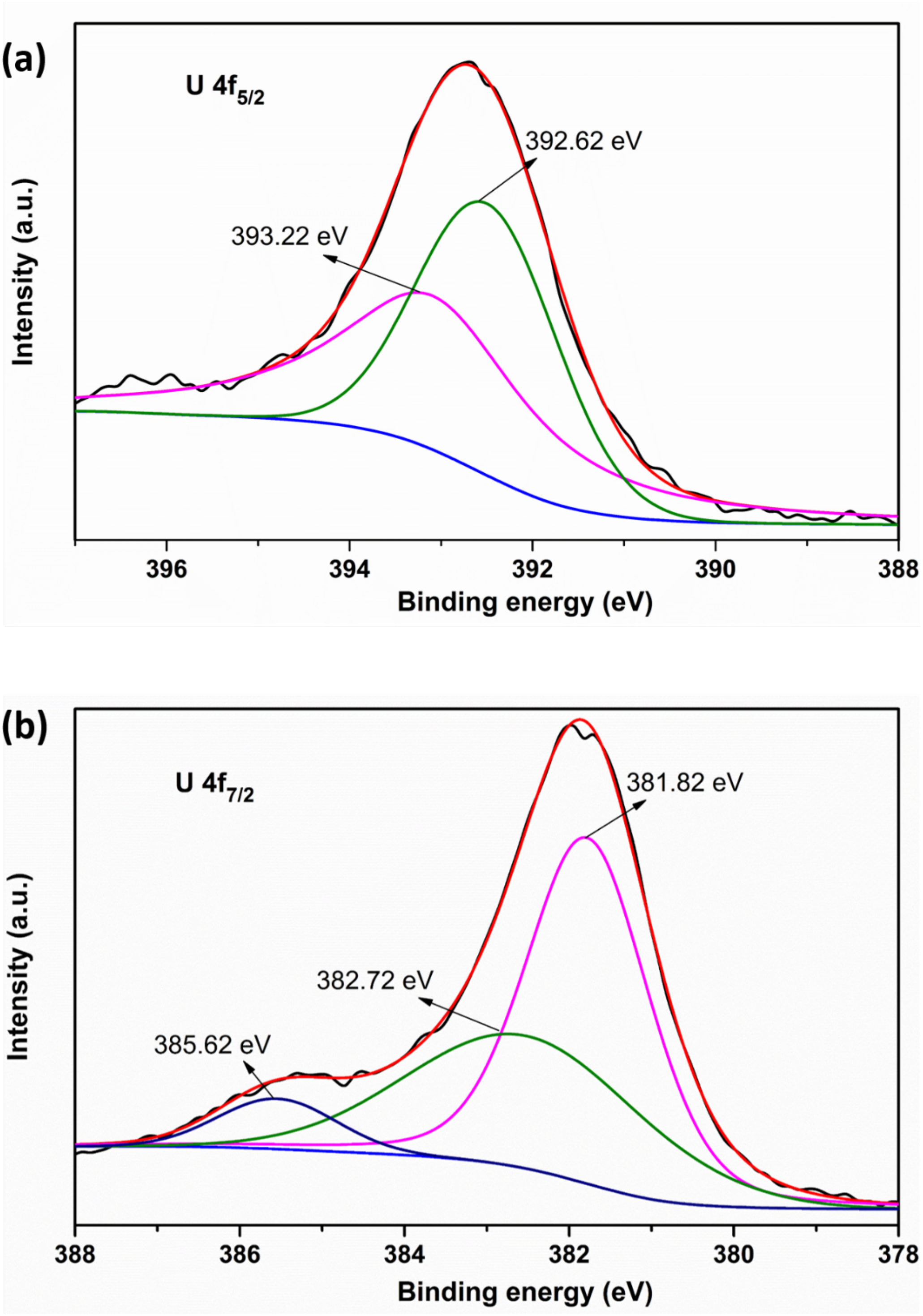

Figure S15. High-resolution XPS spectra of the two U*4f* peaks of the U-uptake hydrogel.

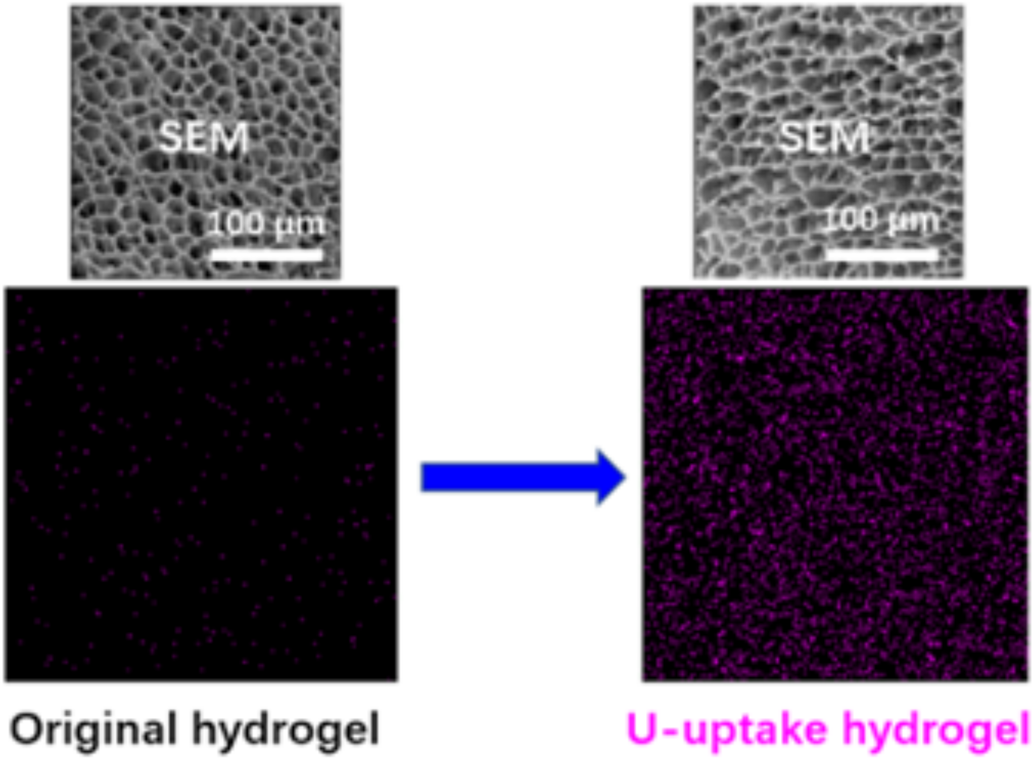

Figure S16. Cross-section SEM image of the U-uptake hydrogel and the corresponding EDS mapping of uranium element.

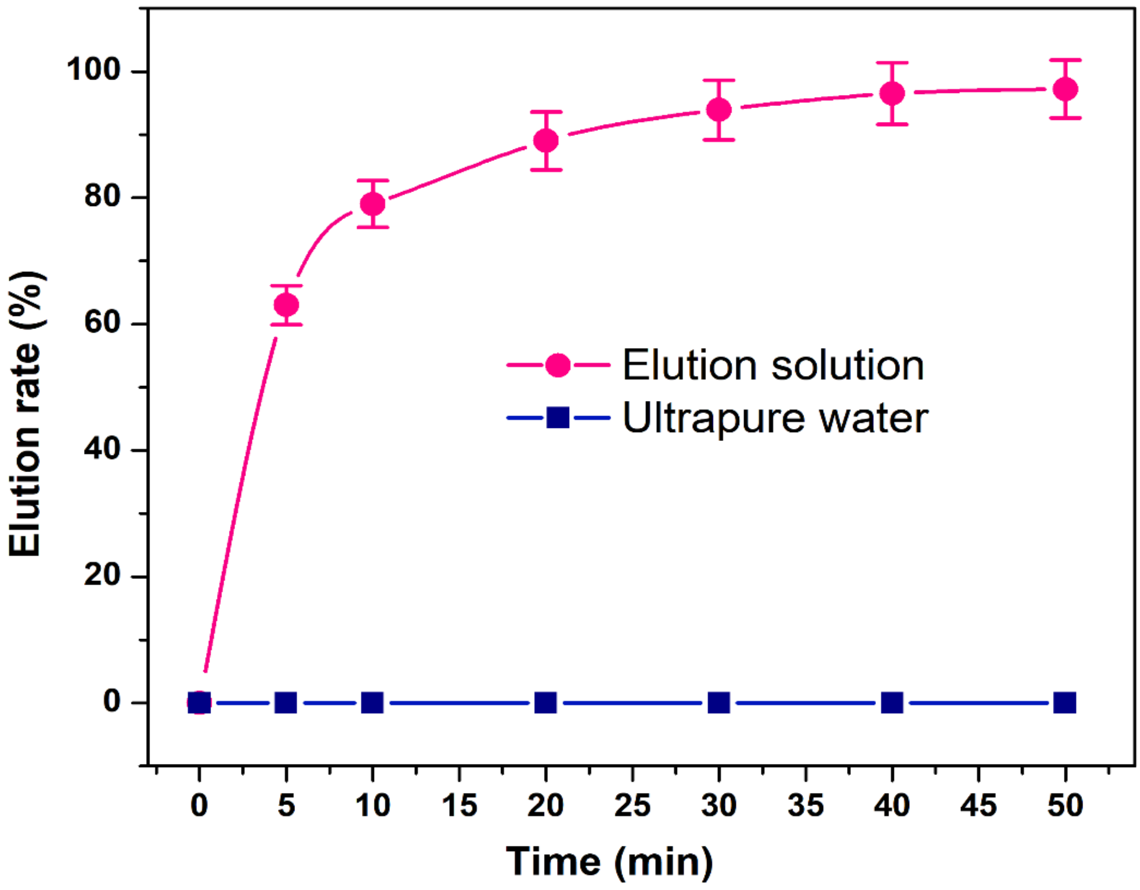

Figure S17. Uranium desorption kinetics of the directional channel hydrogel in the elution solution (n = 3). Data are shown ss means ± SD.

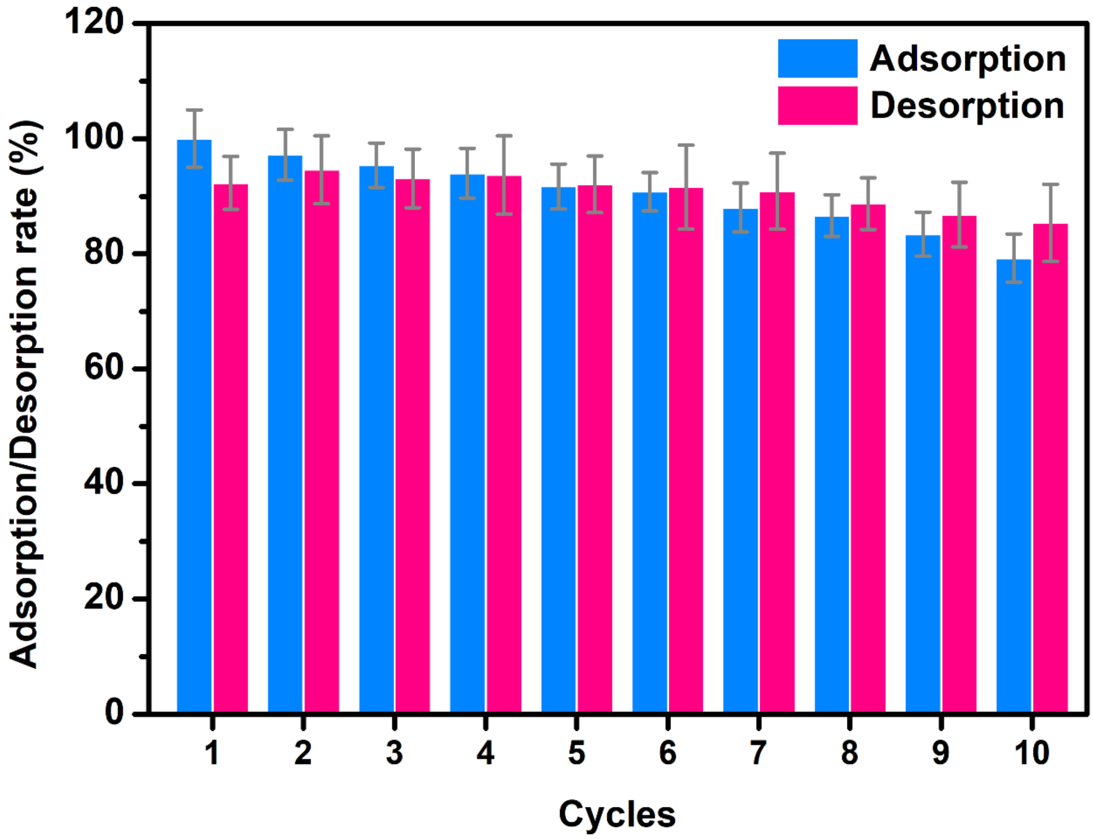

Figure S18. The uranium adsorption capacities (blue columns) and recovery rate of elution (red columns) in ten adsorption-desorption cycles (*n* = 3). Data are shown ss means ± SD.

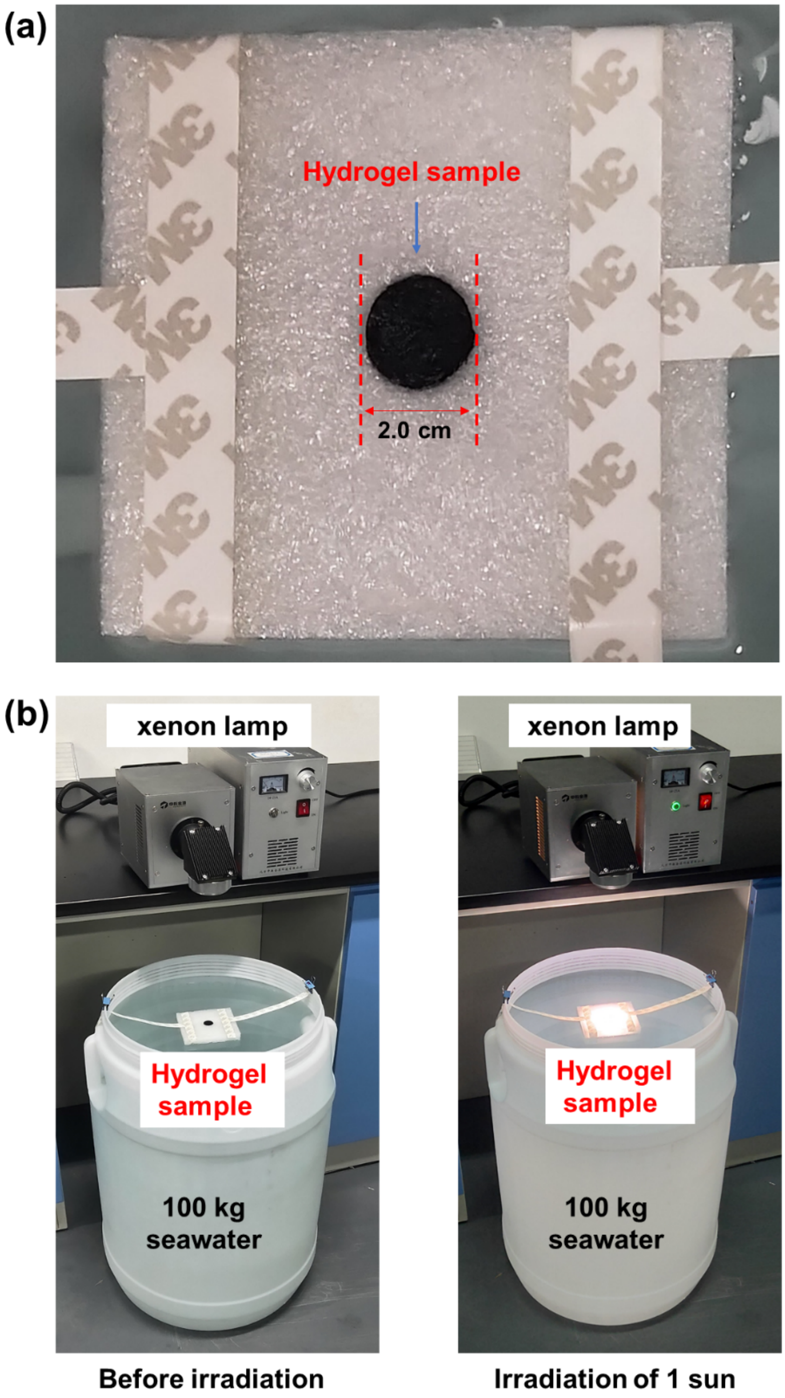

Figure S19. Equipment used for simulated sunlight irradiated uranium extraction from natural seawater.

Supporting Tables

Table S1. Comparison of the evaporation rate between directional channel hydrogels fabricated with different solid content under one sun within seawater.

Sample	Energy efficiency (%)	Evaporation rate (kg m^{-2} h^{-1})
Solid content f 7%	The hydrogel cannot exist stably in seawater	
Solid content f 8%	88.3 ± 1.53	2.18 ± 0.32
Solid content f 9%	90.7 ± 1.65	2.86 ± 0.28
Solid content f 10%	90.5 ± 1.82	1.91 ± 0.34
Solid content f 12%	91.6 ± 1.47	1.26 ± 0.19

Table S2. Concentration of U(VI) and co-existing metal ions in seawater and 100× seawater.

Element	Con. In natural SW/ppb	Con. In 100× SW (calculated)/ppb	Ions
U	3.1	310	U(VI)
V	2.3	230	V(V)
Fe	1.8	180	Ni^{2+}
Co	2.7	270	Cu^{2+}
Ni	3.1	310	Fe^{3+}
Cu	1.4	140	Zn^{2+}
Na	9.01×10^{6}	9.01×10^{6}	Na^{+}
K	0.63×10^{6}	0.63×10^{6}	K^{+}
Ca	0.74×10^{6}	0.74×10^{6}	Ca^{2+}
Mg	1.13×10^{6}	1.13×10^{6}	Mg^{2+}

Table S3. Comparison among uranium adsorption capacities of adsorbents from natural seawater in the past two decades (PAO and AO are the abbreviations of poly(amidoxime) and amidoxime, respectively).

Absorbents	Year	Q_U (mg g^{-1})	Time (day)	Average rate (mg g^{-1} day^{-1})	Ref.
Poly(amidoxime) composite hydrogel	2020	6.21	42	0.148	[4]
Amidoxime Aerogels	2020	9.29±0 59	30	0.310	[5]
poly(amidoxime) porous	2020	9.35± 0.47	35	0.267	[6]
Zn^{2+}-PAO hydrogel membrane	2020	9.23	28	0.330	[7]
PAO hydrogel-sponge hybrid	2019	1.87	56	0.033	[8]
PAO Hydrogel membrane	2019	4.87	28	0.174	[1]
AO-based fibers	2020	17.57	90	0.195	[9]
AO-based fibers	2020	10.31	35	0.295	[10]
AO-based fiber	2019	11.5	90	0.128	[11]
AO-based nanofiber	2019	9.59	30	0.320	[12]
AO-based nanofiber	2018	8.7	56	0.155	[13]
polyethylene fiber	2017	10	140	0.071	[14]
olyethylene fiber	2017	7.4	96	0.077	[14]
AO-based fiber	2016	6.9	56	0.123	[15]
polyethylene fiber	2015	4.15	65	0.064	[16]
AO-based fiber	2014	3.3	56	0.059	[17]
AO-based fiber	2013	2	30	0.067	[18]
AO-based fiber	2010	1.5	30	0.05	[19]

Supporting References

[1] C. X. Ma, J. X. Gao, D. Wang, Y. H. Yuan, J. Wen, B. J. Yan, S. L. Zhao, X. M. Zhao, Y. Sun, X. L. Wang, N. Wang, *Adv. Sci.* **2019**, *6,* 1900085.

[2] S. L. Zhao, Y. H. Yuan, Q. H. Yu, B. Y. Niu, J. H. Liao, Z. H. Guo, N. Wang, *Angew. Chem. Int. Edit.* **2019**, *58*, 14979.

[3] Q. H. Yu, Y. H. Yuan, L. J. Feng, T. T. Feng, W. Y. Sun, N. Wang, *Angew. Chem. Int. Edit.* **2020**, *59*, 15997.

[4] J. X. Gao, Y. H. Yuan, Q. H. Yu, B. J. Yan, Y. X. Qian, J. Wen, C. X. Ma, S. H. Jiang, X. L. Wang, N. Wang, *Chem. Commun.* **2020**, *56*, 3935.

[5] S. Shi, B. C. Li, Y. X. Qian, P. P. Mei, N. Wang, *Chem. Eng. J.* **2020**, *397*, 125337.

[6] S. Shi, Y. X. Qian, P. P. Mei, Y. H. Yuan, N. Jia, M. Y. Dong, J. C. Fan, Z. H. Guo, N. Wang, *Nano Energy* **2020**, *71*, 104629.

[7] B. J. Yan, C. X. Ma, J. X. Gao, Y. H. Yuan, N. Wang, *Adv. Mater.* **2020**, *32*, 1906615.

[8] D. Wang, J. N. Song, S. Lin, J. Wen, C. X. Ma, Y. H. Yuan, M. Lei, X. L. Wang, N. Wang, H. Wu, *Adv. Funct. Mater.* **2019**, *29*, 1901009.

[9] X. Xu, L. Xu, J. X. Ao, Y. L. Liang, C. Li, Y. J. Wang, C. Huang, F. Ye, Q. N. Li, X. J. Guo, J. Y. Li, H. T. Wang, S. Q. Ma, H. J. Ma, *J. Mater. Chem. A* **2020**, *8*, 22032.

[10] Z. Li, Z. Q. Yu, Y. D. Wu, X. L. Wu, Y. Wan, Y. H. Yuan, N. Wang, *Chem. Eng. J.* **2020**, *390*, 124648.

[11] X. Xu, H. J. Zhang, J. X. Ao, L. Xu, X. Y. Liu, X. J. Guo, J. Y. Li, L. Zhang, Q. N. Li, X. Y. Zhao, B. J. Ye, D. L. Wang, F. Shen, H. J. Ma, *Energ. Environ. Sci.* **2019**, *12*, 1979.

[12] Y. H. Yuan, S. L. Zhao, J. Wen, D. Wang, X. W. Gu, L. L. Xu, X. L. Wang, N. Wang, *Adv. Funct. Mater.* **2019**, *29*, 1805380.

[13] D. Wang, J. A. Song, J. Wen, Y. H. Yuan, Z. L. Liu, S. Lin, H. Y. Wang, H. L. Wang, S. L. Zhao, X. M. Zhao, M. H. Fang, M. Lei, B. Li, N. Wang, X. L. Wang, H. Wu, *Adv. Energy Mater.* **2018**, *8*, 1802607.

[14] L. J. Kuo, H. B. Pan, C. M. Wai, M. F. Byers, E. Schneider, J. E. Strivens, C. J. Janke, S. Das, R. T. Mayes, J. R. Wood, N. Schlafer, G. A. Gill, *Ind. Eng. Chem. Res.* **2017**, *56*, 11603.

[15] S. Brown, Y. F. Yue, L. J. Kuo, N. Mehio, M. J. Li, G. Gill, C. Tsouris, R. T. Mayes, T. Saito, S. Dai, *Ind. Eng. Chem. Res.* **2016**, *55*, 4139.

[16] S. Das, W. P. Liao, M. F. Byers, C. Tsouris, C. J. Janke, R. T. Mayes, E. Schneider, L. J. Kuo, J. R. Wood, G. A. Gill, S. Dai, *Ind. Eng. Chem. Res.* **2016**, *55*, 4303.

[17] J. Kim, C. Tsouris, Y. Oyola, C. J. Janke, R. T. Mayes, S. Dai, G. Gill, L. J. Kuo, J. Wood, K. Y. Choe, E. Schneider, H. Lindner, *Ind. Eng. Chem. Res.* **2014**, *53*, 6076.

[18] M. Picard, C. Baelden, Y. Wu, L. Chang, A. H. Slocum, *Nucl. Technol.* **2014**, *188*, 200.

[19] M. Tamada, *International Seminar On Nuclear War And Planetary Emergencies—42nd Session, World Scientific* **2010**, 243.
